# Towards Perfluoroalkyl and Polyfluoroalkyl Substance (PFAS)-Free Energy Harvesting: Recent Advances in Triboelectric Nanogenerators for Sports Applications

**DOI:** 10.3390/mi16030313

**Published:** 2025-03-07

**Authors:** Mónica P. S. Ferreira, Inês Ferreira, Vânia Pais, Liliana Leite, João Bessa, Fernando Cunha, Raúl Fangueiro

**Affiliations:** 1 Fibrenamics—Institute for Innovation in Fiber-Based Materials and Composites, University of Minho, Campus de Azurém, 4800-058 Guimarães, Portugal; 2 Centre for Textile Science and Technology (2C2T), University of Minho, Campus de Azurém, 4800-058 Guimarães, Portugal

**Keywords:** triboelectric nanogenerator (TENG), sports applications, perfluoroalkyl and polyfluoroalkyl substances (PFAS), PFAS-free

## Abstract

Triboelectric nanogenerators (TENGs) can convert the mechanical energy of physical activities into electricity. This is particularly useful in sports applications, where physical activity can power devices such as wearables that can provide real-time feedback on athletes’ performance or health. To work, a TENG usually needs tribopositive and tribonegative materials. Currently, the vast majority of TENGs use materials containing perfluoroalkyl and polyfluoroalkyl substances (PFAS) as tribonegative materials. However, these substances pose risks to humans and the environment, which has led the European Union to consider restrictions on these compounds. For this reason, PFAS-free alternatives, such as polydimethylsiloxane (PDMS) and MXenes, need to be better explored to replace PFAS materials while aiming to achieve equal efficiency. This review will explore some of the recent advances that have been developed in the field of PFAS-free TENGs, with an emphasis on sports applications.

## 1. Introduction

With the rise of the Internet of Things and data analytics, there is a greater demand for a wider range of mobile wearable electronics, especially in the area of sports analytics. Sports monitoring and data analysis can provide a better understanding of an athlete’s health and performance, resulting in better training outcomes [1]. Currently, most sports monitoring is conducted with high-speed cameras and referee observations, which can lead to biased or unfair decisions [2]. Although the use of wearables in sports is growing, the devices available on the market are mainly designed to improve performance [3], sometimes neglecting other critical aspects such as health monitoring and injury prevention. These devices, which include gyroscopes, accelerometers, and magnetometers—sensors that measure motion and orientation—rely on large batteries or supercapacitors powered by fossil fuels [4], which present environmental and practical problems, such as frequent recharging and limited flexibility. Consequently, there is a pressing need for wearable devices that combine self-powered operation with real-time data capabilities for comprehensive sports monitoring.

To address this problem, sustainable and efficient energy sources such as piezoelectric [5], thermoelectric [6], electromagnetic [7], and triboelectric nanogenerators [8] are gaining popularity as low-carbon alternatives. Triboelectric nanogenerators (TENGs) are notable for their flexibility, lightweight design, low cost, and ability to generate a high electrical output. Furthermore, they are simple to build and may be made from a variety of materials [5,9]. Unlike other energy sources, TENGs can convert mechanical energy generated during physical activities, such as running, jumping, or swinging, into usable electrical energy. This makes them perfect for self-powered sports gear that harvests energy, monitors motion, and delivers real-time performance feedback during training or competition. Several studies have demonstrated the successful integration of TENGs into wearable systems designed specifically for sports applications, such as garments or footwear [10,11,12], and training equipment [13,14,15].

The electrical output of TENGs is influenced by the materials used (one tribonegative and one tribopositive) and the roughness of their surfaces, which affect friction and contact area, ultimately determining the output. Tribonegative layers are often made of polytetrafluoroethylene (PTFE) and polyvinylidene fluoride (PVDF), whereas triboposite layers are often made of polyamide and aluminium. The decision of which materials should be used is based on their positions in the triboelectric series, which ranks materials by their ability to receive (tribonegative) or lose (tribopositive) electrons [16]. However, due to their toxicological profiles, certain commonly used tribonegative materials—polyfluoroalkyl substances (PFAS)—are currently under scrutiny in the European Union (EU). The European Chemical Agency (ECHA) emphasizes the need to restrict the “manufacture, placing on the market and use of PFASs”, highlighting that these substances “are, or ultimately transform into, persistent substances” that result in “irreversible environmental exposure and accumulation”. Their solubility and mobility have led to contamination of water and soil, with removal being “very difficult and extremely costly”. Some PFASs are considered “toxic and/or bioaccumulative substances”, posing serious risks to human health and the environment. “Without taking action, their concentrations will continue to increase, and their toxic and polluting effects will be difficult to reverse” [17]. Hence, it is imperative to find more sustainable alternatives.

In response to these environmental challenges, research is shifting to eco-friendly alternatives to PFAS-based materials. Promising tribonegative candidates include polydimethylsiloxane (PDMS) [12], cellulose-based materials [18], and MXenes [19]. Each alternative has unique advantages: PDMS is elastic and of easy production at low cost [20]; cellulose-based materials are low cost and biodegradable, though they require modification to enhance triboelectric performance [21]; and MXenes have tunable composition and surface properties, which allows for a diverse range of electrical and mechanical properties [19].

In addition to sustainability, another important feature of TENGs for sports applications is their biocompatibility. If used in contact with the skin, TENGs should be made of non-toxic, non-irritable materials that are safe for humans.

This review aims to raise awareness of the use of PFAS by highlighting sports-related research that avoids their use while focusing on material breakthroughs, device designs, and their impact on performance monitoring and athlete safety. The next sections will cover the fundamental principles of TENGs, commonly used PFAS-containing materials, and emerging PFAS-free materials, as well as recent advancements in sports applications.

## 2. Working Mechanism of TENGs

**Figure 1:**
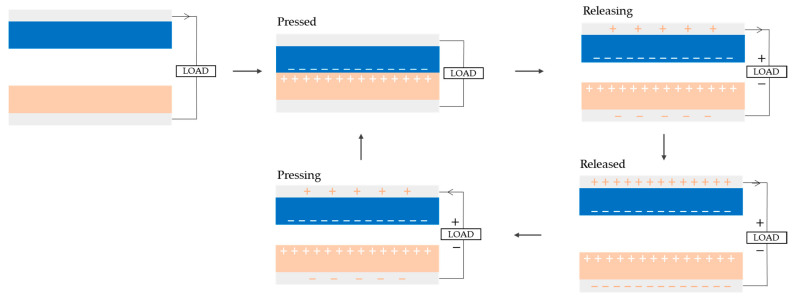
Mechanism for generating electricity in a triboelectric nanogenerator, operating in the contact separation (CS) mode, through contact electrification and electrostatic induction. Adapted with modifications from [28], licensed under the Creative Commons Attribution 3.0 Unported License (https://creativecommons.org/licenses/by/3.0/, accessed on 3 December 2024).

The working mechanism of TENGs is based on triboelectricity, a phenomenon that, while not entirely understood, involves the interaction between two materials: one that is prone to giving electrons, and one that is prone to accepting them [22]. Triboelectrification can occur between various material interfaces, including materials in different physical states: solid–solid [12], liquid–liquid [23], solid–liquid [24], and liquid–gas [25]. When these materials come into contact with each other, two effects take place: contact electrification and electrostatic induction (Figure 1). Through contact electrification, when the two materials come into contact, electrostatic charges are generated and stored on the surface of the dielectric material. At this stage, both materials have equivalent but opposite charges [26]. As the dielectric changes position, electrostatic induction triggers the transfer of electrons between them. Triboelectrification can occur in a variety of ways, resulting in three operational modes: contact separation (CS), lateral sliding (LS), and freestanding (FS). The first two can operate with one or two electrodes, while the FS mode normally requires two electrodes [22]. The single-electrode mode was developed as an alternative to the double-electrode configuration, which is typically less feasible due to the necessity for lead-out wires and is, hence, limited for usage in self-powered sensing applications [27].

**Figure 2:**
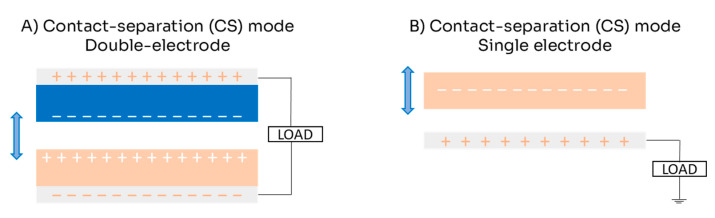
Scheme for contact separation (CS) mode of triboelectric nanogenerator: (**A**) double-electrode configuration; (**B**) single-electrode configuration.

The CS mode is the simplest working mode; it is based on a vertical charge separation movement. This mechanism can operate with either one or two electrodes (see Figure 2). In the double-electrode configuration, two dielectric materials face each other, each with a metallic layer attached to serve as electrodes (Figure 2A). When they come into contact, their surfaces generate opposing charges; when they separate, a potential difference is induced between the two electrodes, causing electrons to move from one electrode to the other (see Figure 1). When the materials come into contact again, the electrons return to their original positions. In the single-electrode configuration, a single dielectric material interacts with a fixed electrode (Figure 2B). When the dielectric comes into contact with the electrode, it generates triboelectric charges on its surface and induces opposite charges on the electrode [22,26]. As the dielectric separates, an electric potential imbalance occurs, allowing electrons to flow from the electrode to the ground. When the materials come together again, electrons flow back from the ground to the electrode [27]. This back-and-forth flow of electrons results in an alternating current (AC) [22].

**Figure 3:**
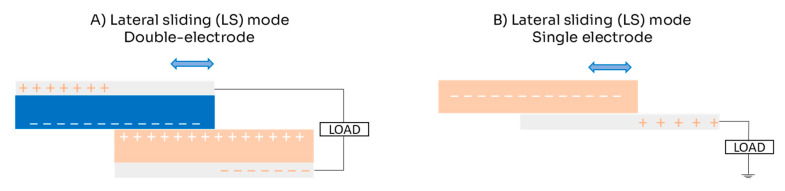
Scheme for lateral sliding (LS) mode of triboelectric nanogenerator: (**A**) double-electrode configuration; (**B**) single-electrode configuration.

The LS mode is similar to the CS mode, but instead of vertical movement, it uses in-plane charge separation movement (which can also include rotational movement). In this mode, two triboelectric layers slide on top of each other, modifying their contact areas but not totally separating (Figure 3). During the sliding motion, opposite charges accumulate in areas where the surfaces do not overlap. Although LS mode functions similarly to CS mode in both single- and double-electrode configurations, one drawback is its vulnerability to mechanical abrasion, which can lead to damage over time [22,26].

**Figure 4:**
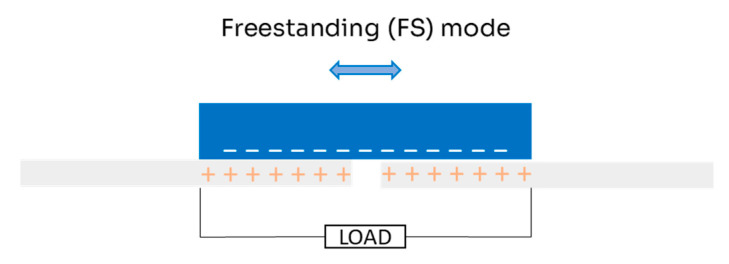
Scheme for freestanding (FS) mode of triboelectric nanogenerator in double-electrode configuration.

The FS mode (Figure 4) was designed to improve on single-electrode TENGs, which typically have a charge transfer efficiency of less than 50%. FS mode, like single-electrode mode, allows for the movement of an object without a direct connection to the TENG [26]. However, several factors need to be considered for this configuration, such as the size of the electrodes, the size of the moving object, and the space between the electrodes. These elements must have dimensions of the same order to ensure an asymmetric charge distribution as the moving object switches between the electrodes, allowing continuous electron flow [29]. A common design in FS mode is rotational TENGs, in which physical contact is unnecessary, which greatly reduces wear [30,31,32,33].

The working modes of TENGs are influenced by several electrical and mechanical parameters, including capacitance, impedance, material thickness, dielectric properties, surface charge density, and the separation gap. Readers interested in a more detailed analysis of these parameters are encouraged to refer to [16].

The majority of TENGs rely on solid–solid triboelectrification, but this often leads to abrasion and wear. Alternative modes—such as solid–liquid, solid–gas, or liquid–gas—may offer solutions to these problems by reducing direct surface friction [22].

## 3. Material Choices for TENGs

**Figure 5:**
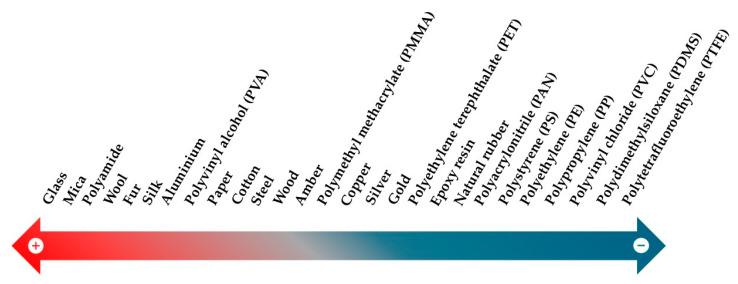
Triboelectric series, which ranks materials according to their tendency to lose (+) and gain (−) electrons.

The triboelectric effect depends on the contact of two materials with distinct tendencies to gain or lose electrons. Hence, material selection is critical to TENG performance. These tendencies are ranked on the triboelectric series (Figure 5), with materials on the positive end being more likely to lose electrons (tribopositive) and those on the negative end being more likely to gain electrons (tribonegative). TENGs often use materials from opposite ends of the triboelectric series to generate a large potential difference, resulting in high output performance. Additionally, low conductivity or insulating materials are often preferred since they can retain charges with ease [22].

Researchers have long employed PFAS materials, notably PTFE, as tribonegative materials due to their ability to hold negative charges. However, these materials cause environmental and health problems, driving the search for PFAS-free alternatives. In contrast, polyamide and metals are commonly utilized as tribopositive materials [34] and do not carry these PFAS-related concerns. These materials are essential to complete the triboelectric pair.

The next subsections will focus on tribonegative materials, discussing the most often used PFAS-containing tribonegative materials, as well as PFAS-free options for more sustainable TENG designs.

### 3.1. PFAS-Containing Materials

Materials from the family of PFAS, such as PTFE, fluorinated ethylene propylene (FEP), and PVDF, are commonly used in TENGs as tribonegative materials. The reason for this is their inherent high negative charge affinity because of their high fluorine content [35], which enhances the electron-gaining capability during the triboelectric effect. In fact, fluorine is the most electronegative element, with five electrons in its 2p orbital [36]. Since the optimal configuration in the 2p orbital is six electrons, the electrons are held very firmly to the nucleus.

Multiple industries [37] favour these materials, not only for their high performance but also due to their thermal stability, chemical resistance, and durability [38]. A significant portion of the published TENG devices rely on PFASs like PTFE or FEP [10,15,39,40,41,42,43,44,45,46,47,48,49] or have been functionalized with PFAS-based components to enhance the tribonegativity of the dielectric layer (see Table 1). Among these, PTFE has consistently exhibited higher performance in voltage, current, and power density [50,51], frequently outperforming other materials. Less common PFAS materials used in TENGs include perfluoroalkoxy (PFA) [52] and ethylene-tetra-fluoro-ethylene (ETFE) [53].

#### Environmental and Health Concerns of PFAS

Despite their advantages, PFASs pose serious environmental and health risks [55,56]. Often referred to as “forever chemicals” due to their resistance to degradation, certain PFASs can persist in the environment, contaminating water supplies and bioaccumulating in organisms [57]. PFASs have been detected in water [55], soil [58], and organisms [59] as a result of their production and use.

Epidemiological studies suggest that increased exposure to certain PFAS is associated with various health problems, including [57]:

- Decreased antibody response;
- Increased risk of certain cancers;
- Altered liver enzyme levels;
- Increased risk of pregnancy-induced hypertension.

Human exposure to PFAS occurs through multiple routes, including [60]:

- Consumption of contaminated food, particularly seafood [61];
- Ingestion of contaminated drinking water, especially near contaminated sites [62];
- Inhalation of indoor air and dust, particularly in environments containing PFAS-treated products such as carpets, water-repellent textiles, and upholstery [63];
- Dermal absorption through skin contact is an emerging concern that has been historically underestimated [64].

Among these routes, food and drinking water are the dominant sources of PFAS exposure [65]. Additionally, PFAS precursors in household products can become airborne and be inhaled. Although dermal exposure has traditionally been considered less significant [65], recent studies suggest that certain PFAS may penetrate the skin at higher levels than previously thought. For example, Ragnarsdóttir et al. [64] used human skin models to assess the absorption rates of 17 different PFAS compounds and found that shorter-chain PFAS were more readily absorbed than longer-chain PFAS, with the highest absorption fraction nearing 60%.

In view of these risks, global regulations are becoming stricter regarding the use of PFAS [17]. Although fluoropolymers like PTFE, PVDF, and FEP are considered chemically stable and non-toxic under normal conditions, their production, processing, and disposal can lead to emissions of low molecular weight PFASs into the environment [66]. While some manufacturers have implemented changes to reduce emissions, concerns about potential harmful effects remain. Furthermore, the fragmentation of PFAS-based materials into microplastics or nanoparticles raises additional concerns, as these particles could enter the environment [67].

To the best of our knowledge, no studies have specifically investigated the health risks associated with PFAS-containing TENGs. Since PFAS-based compounds make up a major fraction of the tribonegative materials used in TENGs, their use raises sustainability concerns, which is leading researchers to look for more environmentally friendly alternatives. Future studies should investigate the possible release of PFAS from TENGs under mechanical stress, UV exposure, and thermal conditions to assess their long-term environmental and health implications.

### 3.2. PFAS-Free Materials

Some PFAS-free materials that have been investigated in TENGs include natural and synthetic polymers, as well as MXenes (Table 2).

A direct comparison of power densities in Table 1 and Table 2 shows that PFAS-based TENGs generally exhibit higher electrical outputs than their PFAS-free counterparts. As noted earlier, this difference may be due to the fact that polyfluoroalkyl materials rank higher in the triboelectric series, allowing a greater charge density difference and, thus, yielding a higher electrical output. However, this material limitation should not restrict future developments using PFAS-free materials. Surface modifications and material doping techniques, as demonstrated in [73], can also result in high electrical outputs, suggesting that alternatives to PFAS materials remain promising for further development. In the next subsections, some alternatives to PFAS are discussed.

#### 3.2.1. Natural Polymers

Natural polymers like cellulose and chitosan are renewable and biodegradable, which makes them ideal as sustainable materials for TENGs. Although they are usually used as tribopositive materials [68], through chemical modifications and specific pairings, their triboelectric polarity can be changed, allowing them to act as tribonegative materials.

Cellulosic materials, for instance, when paired with materials that have more ability to lose electrons, like metals, can behave as tribonegative materials [21]. Kim and colleagues [76] developed a TENG in which cellulose nanofibers were used as a tribonegative material, paired with silver nanowires that served as the electrode and tribopositive layer. They obtained a power output of 693 mW/m^2^ at 10 MΩ, an open-circuit voltage of 21 V, and a short-circuit current of 2.5 μA. Furthermore, introducing new functional groups to cellulosic materials, like fluorinated groups, can significantly enhance their tribonegativitity [18,77]. Cao et al. [18] developed a TENG using cellulose membranes for both triboelectric layers: the tribopositive layer was a cellulose membrane, and the tribonegative layer was a fluorinated cellulose membrane. Both layers were produced via electrospinning, and the device achieved a maximum output voltage of 94 V, a short-circuit current of 8.5 μA, and a power density of 150 mW/m^2^.

Chitosan can also be engineered to behave as a tribonegative material. Researchers have demonstrated that inducing crystalline structures on chitosan films can change their triboelectric polarity [78]. Another approach, by Fang et al. [79], involved the use of tannic acid to modify the surface of chitosan films. Tannic acid interacts with the electron-donating sites on the surface of chitosan, changing its polarity [61]. In this study, the researchers reached a power density of 674 mW/m^2^ at 28 MΩ and an output voltage of 205 V.

#### 3.2.2. Synthetic Polymers

Synthetic polymers are extensively used in the development of TENGs. While some of the most used are PFAS, such as PTFE and FEP, there are others that do not classify as PFAS, such as polydimethylsiloxane (PDMS) and Kapton [35]. Other examples of synthetic polymers, not classified as PFAS, that have been used in TENGs include polyethylene terephthalate (PET) [80], polystyrene (PS) [81], and polypropylene (PP) [72].

As previously said, among synthetic non-PFAS polymers, PDMS is the most popular due to its durability, flexibility, and non-toxicity [82], properties that make it ideal for applications such as wearables and e-skins. However, PDMS does not achieve the high triboelectric outputs of PTFE. To improve PDMS’s triboelectric output, studies are focusing on surface modifications and incorporation of dielectric particles (e.g., BaTiO_3_ and SiO_2_) [83,84,85] and carbon-based materials (e.g., graphene and carbon nanotubes) [86,87]. Despite these efforts, there are still some concerns in these approaches, like the toxicity of some dielectric particles [88] and the dispersion of carbon-based materials in organic solvents, which often leads to aggregation of the particles that negatively affect triboelectric performance [87].

PS is another polymer that ranks relatively high in the triboelectric series as a tribonegative material. However, as noted by Mudgal et al. [81], its use in TENGs is not very common. These researchers designed a TENG using PS (developed by a drop-cast method) as the primary friction layer, with aluminium as the other layer. The TENG generated a V_oc_ of 140 V, an I_SC_ of 6 μA, and a power density of 80 mW/m^2^, results that suggest the potential of this underexplored material [81].

Just like in the case of natural polymers, there are synthetic polymers that do not necessarily behave as tribonegative but that can be tailored to behave as so. An example is polymethyl methacrylate (PMMA). Although it is positioned in the centre of the triboelectric series and is usually used as a tribopositive material, its polarity can be tailored, as demonstrated by Busolo et al. [89]. The authors used the polarity of the voltage during the electrospinning of PMMA fibres to tailor the surface potential of the fibres: a positive voltage polarity resulted in tribonegative PMMA; a negative voltage polarity resulted in tribopositive PMMA. A TENG developed with the tribonegative PMMA resulted in a peak V_oc_ of 2.9 V, an I_SC_ of 67.7 nA, and a maximum average output of 46.6 nW at 40 MΩ. While this TENG was outperformed by a TENG developed with the tribopositive PMMA, these results demonstrate the potential of manipulating polymers that are not normally suitable for TENGs [89].

There are also some emerging biodegradable synthetic polymers like polycaprolactone (PCL). For instance, Parandeh et al. [90] used a PCL/graphene oxide (GO) electrospun membrane as a tribonegative material in a TENG, where cellulose paper was used as an electron donor. The TENG achieved a maximum open circuit voltage of 120 V, short circuit current of 120 V, and load power of 116 µW. These results showcase how environmentally friendly and low-cost materials can be used to develop TENGs.

#### 3.2.3. MXenes

MXenes are a class of two-dimensional materials comprising transition metal carbides, nitrides, and carbonitrides [91] that, due to their surface functional groups, offer tunable electronic properties [92]. By modifying their surface chemistry, MXenes can enhance electron transfer and the contact area, allowing for tailored triboelectric outputs [92]. Among the various types of MXenes, Ti_3_C_2_T_x_ is the most widely studied and used [93]. Dong and colleagues [19], for example, demonstrated that Ti_3_C_2_T_x_ MXenes can be triboelectrically more negative than PTFE.

Although MXenes are relatively cheap, abundant, and have good mechanical properties [92], there are some questions regarding their surface stability, processing techniques, and long-term performance [92,94]. For example, MXenes are usually synthesized with fluoride-based etchants, which can be toxic and harmful to the environment [92,94]. Consequently, although MXenes can be a viable alternative to PFAS, there is a risk of transferring the environmental burden from one type of fluorine-based material to another. Green synthesis strategies are, therefore, needed to synthesize more environmentally friendly MXenes [94]. Some examples include using green solvents, such as ionic liquid, deep eutectic solvents, and dimethyl isosorbide, and green synthesis techniques, such as microwave treatment, in situ etching, and selective plasma etching, which avoid harmful chemicals and reduce energy consumption [95].

## 4. PFAS-Free TENGs for Sports Applications

In sports applications, comparing PFAS-containing and PFAS-free tribonegative materials reveals important performance distinctions that require thoughtful evaluation. As previously discussed, conventional PFAS materials, such as PTFE, have long dominated this field because of their strong electron-attracting characteristics, resistance to degradation, and excellent mechanical resilience [38]. Nonetheless, emerging research into PFAS-free alternatives has significantly reduced the performance discrepancy while also addressing important sustainability problems. Some PFAS-free materials may need surface modifications, composite integration, or specific designs to obtain comparable performance. Some examples of specific design adaptations include hierarchical microstructured surfaces that maximize effective contact area [11] and composite tribonegative layers with conductive additives [96].

Some of the desired characteristics of TENGs for sports applications include wearability, flexibility [42], stretchability [97], self-healability [2], and conformability to the skin [98] to ensure comfort and long-term performance. Researchers have explored various material strategies to enhance these properties, such as using PDMS for improved flexibility and stretchability [99]. Additionally, the integration of nanostructured surfaces has been shown to improve the mechanical adaptability of TENGs [100]. Despite these advancements, challenges remain in optimizing PFAS-free materials to balance mechanical resilience, environmental stability, and electrical output, particularly under real-world conditions such as sweating, repeated movements, and prolonged wear.

The next sections will examine PFAS-free TENGs for a variety of sports-related applications.

### 4.1. Wearables and e-Skins

The integration of TENGs into wearables and electronic skins (e-skins) is revolutionizing sports monitoring, enabling improvements in training and real-time monitoring of athletes’ health and performance. In addition, they are also improving sports data and revenues, as sports analytics seem to lead to greater fan engagement [101]. Incorporating TENGs into sports equipment makes it possible to detect mechanical stimuli during physical activities without needing an external power supply.

**Figure 6:**
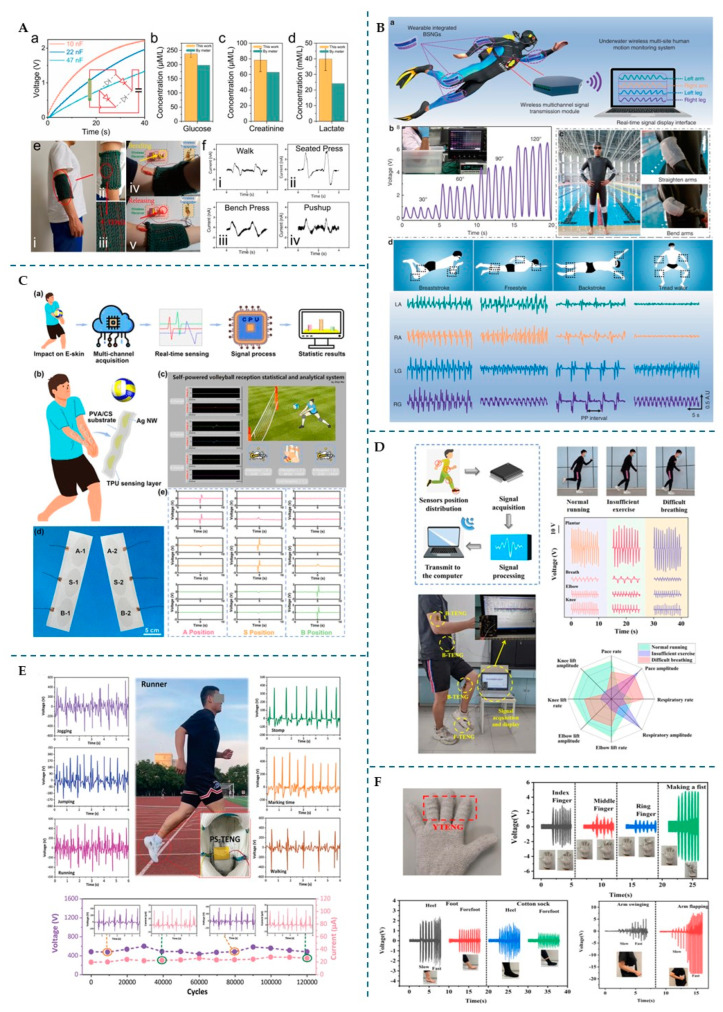
TENG-based wearables and e-skins for sports applications. (**A**) Fabric-TENGs (F-TENGs) for (**b**–**d**) biosensing (glucose, creatinine, and lactate), (**e**) smart clothing, and (**f**) body motion monitoring. Reprinted from [103], Copyright (2022), with permission from Elsevier. (**B**) Underwater motion monitoring system with (**a**) bionic stretchable nanogenerator (BSNG) for (**b**) elbow at different curvature motion, (**c**) joint motion, and (**d**) swimming stroke analysis (left (LA)/right (RA) arm and left (LG)/right (RG) leg). Adapted from [97], licensed under the Creative Commons Attribution 4.0 Unported License (https://creativecommons.org/licenses/by/4.0/, accessed on 17 December 2024). (**C**) (**a**,**b**,**d**) E-skin arrays for volleyball reception analysis, (**e**) tracking impact at different positions, and (**c**) real-time signals. Reprinted with permission from [98]. Copyright 2021, American Chemical Society. (**D**) Application of a body-TENG (B-TENG) and a foot-TENG (F-TENG) for analysing normal running, insufficient exercise, and breathing difficulties. Reprinted from [11], Copyright (2024), with permission from Elsevier. (**E**) Polydimethylsiloxane/silanized graphite oxide-based TENG (PS-TENG) for monitoring various athlete motions (jogging, stomping, jumping, marking time, running, walking). Adapted with modifications from [12], licensed under the Creative Commons Attribution 4.0 Unported License (https://creativecommons.org/licenses/by/4.0/, accessed on 17 December 2024). (**F**) Yarn-TENG (YTENG) and a fabric-TENG (FTENG) for detecting finger, heel, forefoot (with and without cotton socks), and arm motions. Adapted with permission from [69]. Copyright 2021 American Chemical Society.

Wearables, such as smart textiles, wristbands, and insoles, are portable devices integrated into clothing or accessories that monitor physical activity or health parameters [102]. Wearables provide athletes the means to record performance data, movement patterns, and biometric signals, thus helping to prevent injuries and improve performance [102]. On the other hand, e-skins are designed to adhere directly to human skin and allow real-time surveillance of physiological and physical signals. To promote comfort and safety during intimate skin contact, they must be elastic, flexible, breathable, and, ideally, antibacterial [98]. E-skins can detect small movements, stretches, and pressures on the body, making them particularly useful for monitoring sports performance and health. Figure 6 highlights examples from the literature of TENG-integrated wearables and e-skins designed for sports applications.

#### 4.1.1. Athletics

Several PFAS-free TENGs have already been developed for sports monitoring in athletics. For example, Zheng T. et al. [68] developed a single-electrode nitrocellulose-based triboelectric sensor (SNC-TES), using nitrocellulose and silicone as tribopositive materials, alongside conductive fabric as the electrode. The SNC-TES was used as an e-skin, attached to the surface below the elbow. The device successfully differentiated between different types of walking and jogging and different jumping heights, achieving an open-circuit voltage (V_oc_) of 225 V. This triboposite layer’s high stretchability (45%), good transmissivity (83% relative to air), and waterproofness (contact angle of 109°) showcased its potential as a wearable tool for performance tracking. The study highlights how TENGs can capture dynamic movement, offering significant insights into gait and activity monitoring.

In another advancement that illustrates how TENGs can be applied for stretchable devices that adapt to the body’s natural movement, Wen Z. et al. [104] developed an e-skin TENG using a wrinkled structure composed of a thin poly(3,4-ethylenedioxythiophene):polystyrene sulfonate (PEDOT:PSS) film blade-coated on polydimethylsiloxane (PDMS). The device was transparent, highly stretchable, and operated in a single-electrode mode using skin for contact electrification. When attached to joints, it effectively monitored human movement, demonstrating a 23 V output increase, depending on the arm bending angle.

Another innovation in wearable sensors came from Tian X. and Hua T. [69], who selected Tencel as the tribopositive material. Tencel ranks slightly higher than cotton on the triboelectric series, and when mixed with chitosan, it gains antibacterial properties. By twisting a Tencel/chitosan blended yarn (as the wrapping yarn) with a silver-coated polyamide conductive yarn (as the core yarn), the researchers developed a single-electrode triboelectric yarn. The yarn was then used as the weft to develop a woven structure, with cotton serving as the warp. The resulting eco-friendly, antibacterial, and breathable sensor was able to monitor with high accuracy finger gestures (when incorporated into a glove) and body movements (when applied to the foot) (Figure 6F). Its washability and durability, even after five washing cycles, emphasize its practical potential for sport wearable textiles.

Huang, L. et al. [11] developed a self-powered motion monitoring system using two different TENGs working simultaneously. The system consisted of a body-monitoring TENG (B-TENG) for tracking torso movements and a foot-based TENG (F-TENG) for monitoring gait. The F-TENG, designed in the vertical CS mode, used aluminium film and PDMS as triboelectric layers, deposited on a 3D-printed substrate to boost the output voltage. In contrast, the B-TENG operated in single-electrode mode, using PDMS as the tribonegative material and human skin as the tribopositive layer. To enhance performance, the PDMS surface was textured with sandpaper (to increase the roughness), and iron powder was added to the electrode to introduce a magnetic medium, which generated a magnetizing current and improved electrical output. The F-TENG was attached to the bottom of the foot and distinguished between different gait patterns, such as walking, jumping, and running (Figure 6D). The B-TENG was placed on the elbow, knee, and abdomen and provided data on joint flexion angles, breathing patterns, and squat depth. This system demonstrated a comprehensive method for analysing movements in real time, offering information on upper and lower body activities.

Maintaining correct posture while running is imperative for athletes, as it can have an impact on performance and reduce the risk of injury. Real-time data on an athlete’s gait and posture can help coaches personalize training and improve performance. To support this question, Yang X. et al. [105] developed a PDMS-/carbon-based TENG (PC-TENG) that monitors long-distance running. The PC-TENG was constructed with a PDMS dielectric layer and two conductive layers comprised of PDMS and carbon, supported by a silica gel layer substrate. This structure imparted elasticity and durability, making the device appropriate as a stretchable insole. The PC-TENG, with a sensitivity of 33.6 µA/MPa, was fitted in shoes to monitor various gait patterns such as walking, running, and jumping. Its capacity to track long-distance motions highlights its potential as a useful tool for athletes to improve their posture and performance.

Similarly, Zhang, S. et al. [12] developed a TENG to monitor running posture. This device’s tribonegative material was a PDMS layer doped with silanized graphite oxide, and the tribopositive material was nylon. The researchers assembled the TENG in CS mode, inserted it into a shoe, and successfully monitored jogging, long jumps, and walking (Figure 6E). This device exhibited a power density of 1780 mW/m^2^ under a 60 MΩ resistance, which further proves the capability of TENG-based sensors to provide valuable insights into running mechanics and posture.

In another advancement, Lin Z. et al. [106] designed a smart insole with two triboelectric sensors, placed at the forefoot and heel, each consisting of a CS TENG atop an elastic air chamber made from latex film. The TENG used a rubber and a copper layer, with another embedded copper layer in the rubber, to reduce undesired signals caused by environmental factors. This sensor monitored various gait patterns by analysing the time difference between the forefoot and heel contact with the ground. As the forefoot stepped on and off the ground, output peaks were generated, as with the heel. The time difference between these peaks could be critical for subtle gait monitoring. The system was able to discern between walking, running, and jumping, showing promise for sports applications, such as monitoring triple-jump techniques. It tracked the athlete’s speed and timing to determine whether they were optimal for generating forward and height momentum, providing valuable feedback on takeoff and landing accuracy.

Taking a different approach, Chang S. Y., et al. [107] developed a TENG that operated solely with an electrode attached to human skin. This TENG served as both a self-powered warning sensor and a pedometer sensor, capable of harvesting energy from human walking. Its working principle is based on the contact between the shoe and the ground; when the shoe meets the ground, it gains a negative charge, whilst the ground gains a positive charge owing to triboelectrification. When the shoe lifts off the ground, an electrostatic induction occurs, which allows negative charges to flow from the human skin to the ground until they reach a significant distance. When the shoe approaches the ground again, the negative charges flow back. In their tests, the researchers attached a wet silver/silver chloride (Ag/AgCl) electrode to the skin to guarantee safe contact during the measurements. They examined several combinations of footwear and ground, and the combination of thermoplastic rubber shoes and wooden surfaces produced the highest electrical output, with an output voltage of 900 V while walking.

#### 4.1.2. Swimming

Underwater applications of TENGs also show significant promise for sports like swimming. Zou Y. et al. [97], for example, reported a bionic stretchable nanogenerator (BSNG) that mimics the ionic channels of electric eels. The BSNG consists of two layers: the electrification layer, made of PDMS–silicone with controllable fluid channels; and the induction layer, with sodium chloride solution as electrodes. When stretched, the channels open, allowing electrification liquid (deionized water) to flow into the chambers, generating triboelectric charges as negative ions are absorbed onto the silicone surface. This process induces electrostatic charges, creating a potential difference and driving current through the device. Once released, the liquid flows back, reversing the current. The silicone layer provides insulation, allowing the BSNG to function well underwater, though some charges in the water still neutralize the triboelectric charges. The BSNG, inserted in a wristband, was tested on elbows and knees during swimming and differentiated between swimming styles (Figure 6B). It also showed potential for drowning monitoring, acting as a “black box” for swimmers. Additionally, the harvested energy could be stored in a capacitor and power a wireless transmitter for signaling and LED lighting in rescue situations.

#### 4.1.3. Team Sports

TENGs also have potential in team sports, as they can be used to evaluate athletes’ performance. For instance, Yang, H. et al. [73] recently developed a TENG to monitor human posture in basketball. This sensor was built with PET and PDMS/MXene/barium titanate (BaTiO_3_) films as triboelectric layers and operated in CS mode. Attaching the sensor to the knee, foot, and waist allowed for real-time monitoring of human posture, which can provide indirect feedback on dribbling and shooting postures.

Another team sport that can benefit from the use of TENGs is volleyball, as demonstrated by Shi Y. et al. [98]. They developed a single-electrode TENG-based e-skin specifically for this sport, by sandwiching silver nanowires (Ag NW), which operate as electrodes, between two electrospun layers: a TPU top layer and a poly(vinyl alcohol) (PVA)/chitosan bottom layer. The fact that both layers were electrospun contributed to the e-skin’s water vapour permeability. In addition, the bottom layer granted antibacterial properties, an essential feature due to its direct contact with human skin. The e-skin exhibited good elasticity, with the PVA/chitosan layer breaking at 77% strain under a stress of 3.24 MPa, while the TPU layer remained intact until 824% strain. It also demonstrated good recovery from deformation after five cycles of 50% strain. In practical terms, the e-skin was tested as a volleyball reception sensor. When the volleyball impacted both arms during a reception, the e-skin generated two equal output signals (Figure 6C). Following signal processing, these signals supplied real-time data on the player’s reception speed and technique efficacy, allowing for performance analysis that may be used to enhance training.

#### 4.1.4. Respiratory and Vital Monitoring

In sports, monitoring breathing and vital signs not only help improve athletes’ performance but can also help prevent injuries. Zhao Z. et al. [70] described a TENG designed for respiratory monitoring. They developed a textile TENG (t-TENG) from copper-coated polyethylene terephthalate (Cu-PET) yarns for the warp and polyimide (PI)-coated Cu-PET yarns for the weft. Each crisscross point in the woven structure acted as an individual TENG, generating triboelectricity from changes in the contact area caused by bending or tapping. This structure was lightweight and flexible and had lower air resistivity (0.17 ± 0.03 kPa·s/m) than cotton fabric (1.12 ± 0.10 kPa·s/m), making it fitting for wearable applications. The t-TENG was attached to a chest strap to track respiratory activity by detecting abdomen motions during breathing. As the chest expanded and contracted, the t-TENG stretched and relaxed, generating an open-circuit voltage. The authors determined the respiratory rate using the minimum and maximum voltage signals, while the depth was based on the V_oc_ magnitude. The t-TENG was also tested for washability; after three cycles, the current density dropped from 13.78 to 3.52 mA/m^2^, owing to yarn shrinkage and the untwisting of Cu-PET yarns, which affected the contact area. Despite this reduction, t-TENG still worked under different impact velocities, showing promise as a candidate for sports applications.

Fan W. et al. [108] developed a textile-based TENG by combining a conductive yarn with commercial nylon yarn in a full cardigan stitch. This TENG had a sensitivity of 7.84 mV/Pa. Similar to the t-TENG developed by Zhao Z. et al. [70], this TENG had a flexible and breathable structure. However, while Zhao Z. et al.’s TENG was designed to monitor respiratory activity, Fen W. et al.’s device could detect a range of physiological signals. When attached to different body parts, such as the wrist, neck, and finger, the TENG monitored pulse signals. When attached to the chest, it monitored respiration.

In sports performance, tracking an athlete’s health can enhance training and recovery. One promising approach is coupling TENGs with biosensors. Zhao T. et al. [103] introduced a fibre-based TENG (F-TENG) that combines real-time sweat analysis with body motion monitoring to meet this need. To develop the F-TENG, the team functionalized Ecoflex fibres using multiwalled carbon nanotubes (MWCNTs) and polyaniline (PANI), then applied enzymes to the PANI surface and wrapped varnished wires around it. This design enabled the device to track both body motion and sweat composition. When sweat droplets made contact with the F-TENG, an electrical signal containing information regarding glucose, lactate acid, and creatinine levels was generated (Figure 6A). The F-TENG can be woven into textiles, making it ideal for use in sportswear. The triboelectric effect in the F-TENG occurs when movement deforms the varnish, causing it to come into contact with PANI. Furthermore, enzymatic reactions with sweat generate hydrogen peroxide (H_2_O_2_), which decomposes into H^+^ and electrons. These byproducts can increase the triboelectric charge density, resulting in higher current output and allowing the detection of sweat biomarkers to monitor metabolic levels. When compared to commercial meters, the F-TENG showed comparable sensitivity for glucose and creatinine but registered levels of lactate higher than expected. The researchers attributed this to impurities in sweat and lower sensitivity to lactate. They integrated the F-TENG into a sleeve, where it differentiated between different types of motion, such as walking, seated presses, bench presses, and pushups.

### 4.2. Sports Accessories

**Figure 7:**
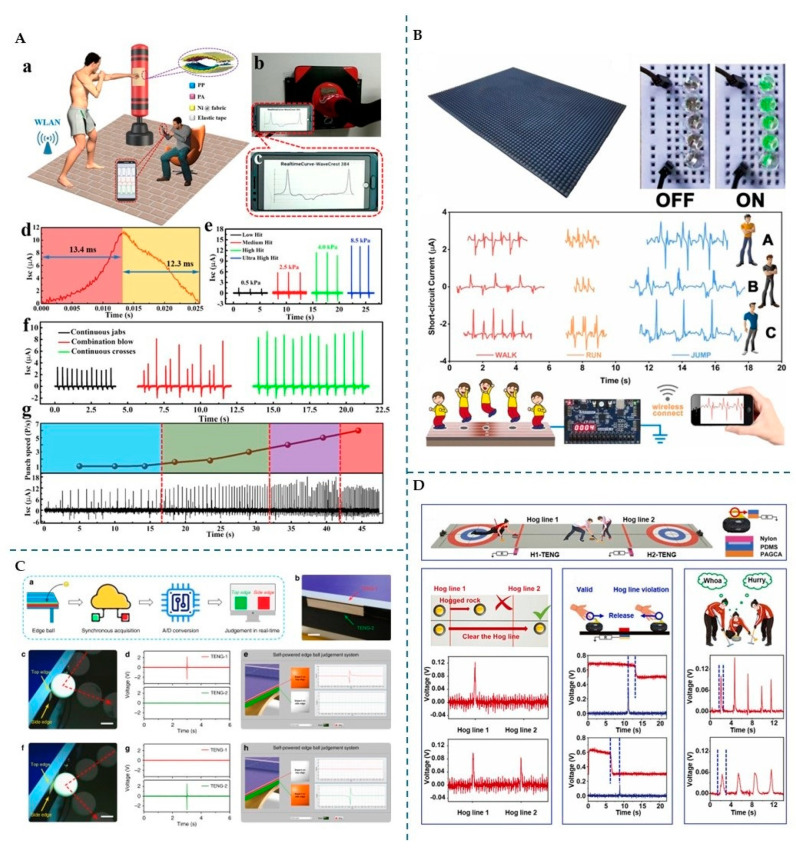
(**A**) (**a**) Self-powered pugilism training monitor (SP-PTM) for (**b**–**d**) tracking training performance, (**e**) punch intensities, (**f**) fist positions, and (**g**) punch speed. Reprinted from [72], Copyright (2019), with permission from Elsevier. (**B**) TENG-based smart track for high-precision exercise monitoring. Reprinted from [109], Copyright (2023), with permission from Elsevier. (**C**) (**a**,**b**) Wood-based-TENG (W-TENG) for edge ball judgment in table tennis, detecting (**c**–**e**) top and (**f**–**h**) side edge balls. Adapted from [14], licensed under the Creative Commons Attribution 4.0 Unported License (https://creativecommons.org/licenses/by/4.0/, accessed on 17 December 2024). (**D**) Self-healing, anti-freezing, and antibacterial nanocomposite hydrogel (PAGCA)-based TENG sensors for smart violation detection in curling, used to judge hogged or cleared stones, detect Hog line violations, and assist in training. Reprinted from [2], Copyright (2024), with permission from Elsevier.

In addition to wearables and e-skins, several PFAS-free accessories have been developed to enhance performance across various sports activities, including table tennis, skiing, boxing, cycling, curling, triple jumping, and fitness. Figure 7 highlights examples from the literature of TENG-integrated accessories designed for sports applications.

#### 4.2.1. Table Tennis

TENGs have shown promise in promoting fairness and performance in table tennis by providing objective measurements to aid decision-making. For instance, Sahu M. et al. [110] developed a single-electrode mode TENG with a triboposite layer made of recycled waste textiles such as jute, cotton, wool, and polypropylene. These fibres were knitted with conductive rubber wire, which served as the electrode and tribonegative layer. Among the fibre combinations tested, the rubber/wool-based TENG demonstrated the highest electrical output, achieving a peak-to-peak voltage of 75 V, a peak-to-peak current of 200 nA, and a charge of 14.7 nC. This TENG was integrated into a table tennis racket, enabling it to determine the quality of balance and hitting frequency. Additionally, it was strategically placed to the edge line of the table tennis court to detect ball contact, thus preventing unfair play resulting from naked eye judgement.

In another study, Luo J. et al. [14] introduced a flexible wood-based TENG designed to minimize unfairness in table tennis. This TENG used balsa wood treated with an alkaline solution to remove lignin and hemicellulose and expose cellulose, which is more tribopositive than the other wood components. A copper electrode was attached to the wood layer and connected to the ground, resulting in a single-electrode TENG. When tested as a self-powered velocity sensor, it displayed a high sensitivity of up to 4.5 m/s and great linearity. Additionally, the TENG was tested as an impact position sensor, with excellent results in detecting the location of a falling object. It was further tested as an edge ball judgement system that discerns between a top edge ball and a side edge ball (Figure 7C). For this application, one TENG was attached to the top edge of the table, while another was placed on the side edge. When the ball struck the top or the side of the table, a corresponding TENG produced an output signal, enabling real-time judgement during table tennis matches.

#### 4.2.2. Winter Sports

Curling was recently recognized as an Olympic winter sport and is receiving more attention. Traditional judgment systems for these games rely on high-speed cameras and thermal sensors, both of which have limitations. To overcome these issues, Tian Z. et al. [2] developed a single-electrode TENG using a hydrogel electrode made of acrylamide with gelatine, carbon nanotubes (CNTs) modified with tannin acid, and silver nanoparticles (AgNPs). The tribonegative material used was PDMS. The hydrogel was designed with anti-freezing properties by replacing its water content with glycerol. It also demonstrated self-healing properties, with an efficiency of 92% for 2 min at 60 °C. Even after self-healing, the TENG retained 95% of its triboelectric properties. The electrical performance remained stable for different temperatures (30 °C and −30 °C). The TENG was assembled as part of a smart violation detection system to accurately detect athletes’ actions, distinguish between valid (two output signals) and invalid scores (a single output signal) during play, and improve training efficiency (Figure 7D). In curling, a hog line violation occurs if the stone is not released before crossing the hog line, which can be hard to judge. To address this, the authors attached a TENG sensor to the curling handle and another TENG under hog line 1. If the output signal of the TENG attached to the handle disappears before the signal from the TENG in hog line 1 appears, the throw is considered valid. Additionally, sensors under the house helped determine which stone was closest to the centre and monitored crashes between stones.

Nanogenerators’ ability to work in harsh environments such as snow can be challenging to fabricate, but Ahmed A. et al. [71] developed a snow-TENG to harvest energy from contact with snow. They used thin 3D-printed PEDOT:PSS films, which acted as the electrode, sandwiched between silicone layers serving as the triboelectric material. To improve the conductivity of the device, the authors added dimethyl sulphate (DMS) to the silicone. The triboelectric layer was patterned to increase surface area contact for better performance. The TENG operated in single-electrode mode through tapping, sliding, and snowfall. In snowfall mode, the TENG could detect snowfall angles and measure snowfall rate and wind speed, as they influence the impact force of the snow particles. The device could be attached to a bicycle wheel for friction energy harvesting. It could potentially be used to monitor cycling activities using bicycles and different human movements if attached to the human body.

In skiing, Yang Y. et al. [13] developed a customizable and flexible TENG using a fused deposition-modeling 3D printer. They used TPU as a friction layer, conductive fabric as electrodes, and silicone rubber film as another friction layer. A thin PU film was used as a waterproof layer due to its compatibility with human skin. The TENG was integrated into ski poles to monitor the frequency and force of the ski pole strikes, as well as step number, thereby improving training sessions. Using 315 samples, it distinguished between three sub-techniques of cross-country skiing with a 100% accuracy rate. When placed in shoe insoles, the TENG could differentiate between different gait patterns, and if attached to human joints, it could distinguish different joint bending angles.

#### 4.2.3. Boxing

Peng F. et al. [54] developed a fabric-based TENG for sensing applications in boxing, where speed and efficiency are crucial. They chose polyamide 6.6 fabric and non-woven polypropylene as triboelectric layers and nickel conductive fabric as electrodes. The TENG was tested as both a pedestrian volume counter and a boxing training monitor. As a pedestrian counter, it monitored pedestrian traffic for a week and showed promise as an energy harvester, with a Voc of 420 V, Isc of 201 µA, and Qsc of 640 nC when stepped on by a 60 kg body. As a boxing training monitor, the TENG was attached to a sandbag, offering a response time of 13.4 ms and a recovery time of 12.3 ms. The TENG showed an increasing current as the hit intensity increased, which allowed for differentiation between low and ultra-high hits (Figure 7A). The sensor also differentiated between different fist positions resulting from different striking techniques. Based on the number of wave crests per second, it was also possible to determine the speed of the punch. The results showed that the sensor could help optimize training sessions.

#### 4.2.4. Triple Jump and Running

Previously, Lin Z. et al. [106] showcased a wearable TENG for monitoring triple-jump gaits. Xu J. et al. [111] introduced a different approach by developing a single-electrode TENG, which is also aimed at enhancing triple jump performance. They designed a take-off board sensor using wood, incorporating grooves filled with urethane rubber as the triboelectric layer and copper wire electrodes. When the athlete steps on the board during the triple jump, the sensor can measure the distance between the front of the athlete’s foot and the take-off line (GAP), which determines whether the take-off is considered a foul. The system showed high accuracy, low cost, and real-time feedback. A smaller GAP value contributes to a larger jumping distance, making it a crucial training metric for athletes. The measurement of the GAP depends on generating an effective voltage in the channel closest to the take-off line. Xu J. and colleagues also tested the effect of athletes’ varying weights on the output voltage, finding no significant difference in the sensor’s performance.

Tracks used for running, sprinting, or jumping are typically made from polyurethane (PU) and ethylene propylene diene monomer (EPDM), which are linked to emissions of volatile organic compounds (VOCs). Recognizing the environmental impact of traditional running tracks, Wu, W. et al. [109] developed a green alternative that functions both as a gait sensor and a training monitor. They developed silicone rubber composites to replace the PU and EPDM elements (frictional layers) using silica as a filler and hydroxyl silicone oil as an additive. For the electrode, the authors developed a conductive layer using carbon black, oil, and silicone rubber. The single-electrode TENG, operating in CS mode, provided information on walking patterns to help improve gait (Figure 7B). Since walking patterns are unique to each individual, the signals generated while stepping on the track can be used to identify and correct bad walking habits.

## 5. Conclusions and Prospects

The utilization of TENGs in the field of sports shows potential as a green, sustainable alternative to current market options. Their high sensitivity and ability to harvest biomechanical energy make them suitable for applications such as motion tracking, self-powered sensors, and performance monitoring. These capabilities highlight TENGs’ potential impact on both training and in-game scenarios, offering benefits for athletes, coaches, and referees.

In this work, the main goal was to draw attention to the potential EU legislation changes—particularly new REACH legislation—that could limit the use of PFAS, a class of substances commonly used in TENG developments. Currently, PFAS-based TENGs display higher power density, particularly in high-output scenarios, which makes them the preferred choice for performance.

However, recent research into PFAS-free materials, including MXenes and silicone- and cellulose-based materials, has demonstrated promising progress. Although PFAS-free TENGs do not yet match the power density and efficiency of PTFE- or FEP-based TENGs, they are approaching these levels. Among the PFAS-free options, MXenes stand out for their potential in TENGs for wearable technology, although some limitations are yet to be overcome [92]. One topic that should be addressed in the usage of MXenes is the use of greener production methods so that we are not attempting to mitigate one environmental problem by creating another. Similarly, cellulose-based materials, such as nano- and micro-cellulose, are also receiving attention due to their easy production and sustainable nature. They might, in the future, have electrical outputs in the same order of magnitude as their synthetic counterparts [112,113]. Considering the potential for a more sustainable energy source from TENGs, the use of environmentally friendly materials should be further investigated.

To close the performance difference between PFAS-free and PFAS-containing TENGs, additional PFAS-free material improvement is required. This includes developing materials that combine the benefits of sustainable substances with enhanced triboelectric properties. Strategies such as surface functionalization, chemical doping, and composite engineering have shown promise in increasing charge density and improving overall triboelectric performance. Additionally, environmental resilience—particularly in the face of humidity, sweat, and wear resistance—remains a key challenge for TENG applications in sports. For instance, when used on the skin, TENGs must withstand sweat; while some reported works test the materials for water-proofness, the TENG output is rarely tested under sweating conditions, an essential area for further research. Additionally, wear resistance, particularly under high-repetition conditions in competitive sports, is critical for TENGs in CS and LS modes, where repeated use leads to abrasion [35]. Alternative, non-solid-based triboelectrification could offer a potential solution by improving long-term reliability and durability [22].

Another important consideration in sports applications is their durability under extended use. Considering that an active person takes about 8000 steps a day, testing for 20,000 cycles or less would be equivalent to the TENG being used for only 2.5 days, which would be insufficient for a sports application. Extensive testing that reflects these real-world applications is necessary to ensure TENGs can meet the demands of a dynamic sports environment.

In summary, PFAS-free TENGs are at a pivotal stage of development. While they have not yet fully matched PFAS-based TENGs in power output and efficiency, they show significant promise. The ongoing development of new materials and technologies will likely lead to more sustainable, efficient PFAS-free TENGs.

## Figures and Tables

**Table 1:** TENGs with PFAS-containing tribonegative materials for sports applications and their electrical outputs.

Application	Triboelectric Materials	Operation Mode	Power Density (mW/cm^2^)	Load Resistance (MΩ)	Ref.
Scoring system for taekwondo	PTFE and PU	CS	0.01	7	[42]
Gait	Chitosan/glycerol and PTFE	CS	0.26	1000	[46]
Smart textile	Fluorinated silk and PA	CS	0.21	10	[48]
Human motion	PLA/chitosan/aloin and TPU/CB-P/PVDF-TrFE	CS	0.0054	10	[45]
Smart saddle	FEP, Cu and Al	CS	0.00013	60	[15]
Human motion	PAN/TiO_2_/PTFE and PA	CS	0.049	4160	[44]
E-textile	PTFE and silane treated cotton	CS	0.6	1	[54]
Human motion	PA66/MWCNTs and PVDF	CS	0.13	24	[49]

CS: contact separation; FEP: fluorinated ethylene propylene; PA: polyamide; PA66/MWCNTs: polyamide 66/multi-walled carbon nanotubes; PAN/TiO_2_/PTFE: polyacrylonitrile/titanium dioxide/polytetrafluoroethylene; PLA: polylactic acid; PTFE: polytetrafluoroethylene; PU: polyurethane; PVDF: polyvinylidene fluoride; TPU/CB-P/PVDF-TrFE: thermoplastic polyurethane/carbon black/poly(vinylidene fluoride-*co*-trifluoroethylene).

**Table 2:** TENGs with PFAS-free tribonegative materials for sports applications and their electrical outputs.

Application	Triboelectric Materials	Operation Mode	Power Density (mW/cm^2^)	Load Resistance (MΩ)	Ref.
Human motion and speech recognition	Nitrocellulose/silicon resin	SE	0.038	100	[68]
Human motion	Tencel/chitosan	SE	0.0016	70	[69]
Breathing	Cu-PET and PI-Cu-PET	CS	0.0033	60	[70]
Human motion	Silicone and snow	SE	0.00002	50	[71]
Pugilism and pedestrian volume	PA and PP	CS	0.09	70	[72]
Human posture	PET and PDMS/MXene/BaTiO_3_	CS	0.24	70	[73]
Gait	PDMS/silanized GO and PA	CS	0.18	60	[12]
Boxing	PDMS/MXene and PA	CS	4.44	40	[74]
Human motion	MXene/PET and NaCl/PVA	CS	0.67	10	[75]

Cu-PET: Cu-polyethylene terephthalate; PA: polyamide; PDMS/MXene/BaTiO_3_: polydimethylsiloxane/MXene/barium titanate; PDMS/silanized GO: polydimethylsiloxane/silanized graphene oxide PET: polyethylene terephthalate; PI-Cu-PET: polyimide-Cu-polyethylene terephthalate; PP: polypropylene.

## References

[1] Ahmadi A., Mitchell E., Richter C., Destelle F., Gowing M., O’Connor N.E., Moran K. (2015). Toward Automatic Activity Classification and Movement Assessment during a Sports Training Session. IEEE Internet Things J..

[2] Tian Z., Zhu Z., Yue S., Liu Y., Li Y., Yu Z.-Z., Yang D. (2024). Self-Powered, Self-Healing, and Anti-Freezing Triboelectric Sensors for Violation Detection in Sport Events. Nano Energy.

[3] Gómez-Carmona C.D., Bastida-Castillo A., Ibáñez S.J., Pino-Ortega J. (2020). Accelerometry as a Method for External Workload Monitoring in Invasion Team Sports. A Systematic Review. PLoS ONE.

[4] Theodoropoulos J.S., Bettle J., Kosy J.D. (2020). The Use of GPS and Inertial Devices for Player Monitoring in Team Sports: A Review of Current and Future Applications. Orthop. Rev..

[5] Bairagi S., Shahid-ul-Islam, Shahadat M., Mulvihill D.M., Ali W. (2023). Mechanical Energy Harvesting and Self-Powered Electronic Applications of Textile-Based Piezoelectric Nanogenerators: A Systematic Review. Nano Energy.

[6] Tabaie Z., Omidvar A. (2023). Human Body Heat-Driven Thermoelectric Generators as a Sustainable Power Supply for Wearable Electronic Devices: Recent Advances, Challenges, and Future Perspectives. Heliyon.

[7] Zhou B., Zhang S., Liu W., Xu F. (2023). A Review of Evaluation, Principles, and Technology of Wearable Electromagnetic Harvesters. ACS Appl. Electron. Mater..

[8] Sun F., Zhu Y., Jia C., Zhao T., Chu L., Mao Y. (2023). Advances in Self-Powered Sports Monitoring Sensors Based on Triboelectric Nanogenerators. J. Energy Chem..

[9] Torres F.G., Gonzales K.N., Troncoso O.P., Corman-Hijar J.I., Cornejo G. (2023). A Review on the Development of Biopolymer Nanocomposite-Based Triboelectric Nanogenerators (Bio-TENGs). ACS Appl. Electron. Mater..

[10] Zhang P., Zhang Z., Cai J. (2021). A Foot Pressure Sensor Based on Triboelectric Nanogenerator for Human Motion Monitoring. Microsyst. Technol..

[11] Huang L., Bu X., Zhang P., Zhang K., Li Y., Wang D., Ding C. (2024). Self-Powered Motion State Monitoring System Based on Combined Triboelectric Nanogenerators for Human Physiological Signal Monitoring and Energy Collection. Microelectron. Eng..

[12] Zhang S., Yang H., Li J. (2023). The Triboelectric Sensor Based on PDMS/SGO for Human Running Posture and Physical Fitness Health Monitoring. Mater. Technol..

[13] Yang Y., Hou X.J., Geng W.P., Mu J.L., Zhang L., Wang X.D., He J., Xiong J.J., Chou X.J. (2022). Human Movement Monitoring and Behavior Recognition for Intelligent Sports Using Customizable and Flexible Triboelectric Nanogenerator. Sci. China Technol. Sci..

[14] Luo J., Wang Z., Xu L., Wang A.C., Han K., Jiang T., Lai Q., Bai Y., Tang W., Fan F.R. (2019). Flexible and Durable Wood-Based Triboelectric Nanogenerators for Self-Powered Sensing in Athletic Big Data Analytics. Nat. Commun..

[15] Hao Y., Wen J., Gao X., Nan D., Pan J., Yang Y., Chen B., Wang Z.L. (2022). Self-Rebound Cambered Triboelectric Nanogenerator Array for Self-Powered Sensing in Kinematic Analytics. ACS Nano.

[16] Trinh V.L., Chung C.K. (2023). Advances in Triboelectric Nanogenerators for Sustainable and Renewable Energy: Working Mechanism, Tribo-Surface Structure, Energy Storage-Collection System, and Applications. Processes.

[17] ECHA Annex XV Restriction Report: Proposal for a Restriction. https://echa.europa.eu/documents/10162/f605d4b5-7c17-7414-8823-b49b9fd43aea.

[18] Cao M., Chen Y., Sha J., Xu Y., Chen S., Xu F. (2024). All-Cellulose Nanofiber-Based Sustainable Triboelectric Nanogenerators for Enhanced Energy Harvesting. Polymers.

[19] Dong Y., Mallineni S.S.K., Maleski K., Behlow H., Mochalin V.N., Rao A.M., Gogotsi Y., Podila R. (2018). Metallic MXenes: A New Family of Materials for Flexible Triboelectric Nanogenerators. Nano Energy.

[20] Li J., Shepelin N.A., Sherrell P.C., Ellis A.V. (2021). Poly(Dimethylsiloxane) for Triboelectricity: From Mechanisms to Practical Strategies. Chem. Mater..

[21] Niu Z., Cheng W., Cao M., Wang D., Wang Q., Han J., Long Y., Han G. (2021). Recent Advances in Cellulose-Based Flexible Triboelectric Nanogenerators. Nano Energy.

[22] Kim W.G., Kim D.W., Tcho I.W., Kim J.K., Kim M.S., Choi Y.K. (2021). Triboelectric Nanogenerator: Structure, Mechanism, and Applications. ACS Nano.

[23] Lu Y., Jiang L., Yu Y., Wang D., Sun W., Liu Y., Yu J., Zhang J., Wang K., Hu H. (2022). Liquid-Liquid Triboelectric Nanogenerator Based on the Immiscible Interface of an Aqueous Two-Phase System. Nat. Commun..

[24] Li X., Zhang D., Zhang D., Li Z., Wu H., Zhou Y., Wang B., Guo H., Peng Y. (2023). Solid-Liquid Triboelectric Nanogenerator Based on Vortex-Induced Resonance. Nanomaterials.

[25] Wang F., Yang P., Tao X., Shi Y., Li S., Liu Z., Chen X., Wang Z.L. (2021). Study of Contact Electrification at Liquid-Gas Interface. ACS Nano.

[26] Niu S., Wang Z.L. (2014). Theoretical Systems of Triboelectric Nanogenerators. Nano Energy.

[27] Akram W., Chen Q., Xia G., Fang J. (2023). A Review of Single Electrode Triboelectric Nanogenerators. Nano Energy.

[28] Kim Y.J., Lee J., Park S., Park C., Park C., Choi H.J. (2017). Effect of the Relative Permittivity of Oxides on the Performance of Triboelectric Nanogenerators. RSC Adv..

[29] Wang Z.L. (2014). Triboelectric Nanogenerators as New Energy Technology and Self-Powered Sensors—Principles, Problems and Perspectives. Faraday Discuss..

[30] Zhang Z., Bai Y., Xu L., Zhao M., Shi M., Wang Z.L., Lu X. (2019). Triboelectric Nanogenerators with Simultaneous Outputs in Both Single-Electrode Mode and Freestanding-Triboelectric-Layer Mode. Nano Energy.

[31] Cheng J., Ding W., Zi Y., Lu Y., Ji L., Liu F., Wu C., Wang Z.L. (2018). Triboelectric Microplasma Powered by Mechanical Stimuli. Nat. Commun..

[32] Zhao M., Nie J., Li H., Xia M., Liu M., Zhang Z., Liang X., Qi R., Wang Z.L., Lu X. (2019). High-Frequency Supercapacitors Based on Carbonized Melamine Foam as Energy Storage Devices for Triboelectric Nanogenerators. Nano Energy.

[33] Zhao C., Zhang Q., Zhang W., Du X., Zhang Y., Gong S., Ren K., Sun Q., Wang Z.L. (2019). Hybrid Piezo/Triboelectric Nanogenerator for Highly Efficient and Stable Rotation Energy Harvesting. Nano Energy.

[34] Davoudi M., An C.Y., Kim D.E. (2023). A Review on Triboelectric Nanogenerators, Recent Applications, and Challenges. Int. J. Precis. Eng. Manuf.-Green. Tech..

[35] Zhang R., Olin H. (2020). Material Choices for Triboelectric Nanogenerators: A Critical Review. EcoMat.

[36] Tantardini C., Oganov A.R. (2021). Thermochemical Electronegativities of the Elements. Nat. Commun..

[37] Glüge J., Scheringer M., Cousins I.T., Dewitt J.C., Goldenman G., Herzke D., Lohmann R., Ng C.A., Trier X., Wang Z. (2020). An Overview of the Uses of Per- And Polyfluoroalkyl Substances (PFAS). Environ. Sci. Process Impacts.

[38] Krafft M.P., Riess J.G. (2015). Selected Physicochemical Aspects of Poly- and Perfluoroalkylated Substances Relevant to Performance, Environment and Sustainability-Part One. Chemosphere.

[39] Meng X.S., Li H.Y., Zhu G., Wang Z.L. (2015). Fully Enclosed Bearing-Structured Self-Powered Rotation Sensor Based on Electrification at Rolling Interfaces for Multi-Tasking Motion Measurement. Nano Energy.

[40] He C., Zhu W., Gu G.Q., Jiang T., Xu L., Chen B.D., Han C.B., Li D., Wang Z.L. (2018). Integrative Square-Grid Triboelectric Nanogenerator as a Vibrational Energy Harvester and Impulsive Force Sensor. Nano Res..

[41] Lu Z., Wen Y., Yang X., Li D., Liu B., Zhang Y., Zhu J., Zhu Y., Zhang S., Mao Y. (2023). A Wireless Intelligent Motion Correction System for Skating Monitoring Based on a Triboelectric Nanogenerator. Electronics.

[42] Sun F., Zhu Y., Jia C., Ouyang B., Zhao T., Li C., Ba N., Li X., Chen S., Che T. (2022). A Flexible Lightweight Triboelectric Nanogenerator for Protector and Scoring System in Taekwondo Competition Monitoring. Electronics.

[43] Wu Z., Zhang B., Zou H., Lin Z., Liu G., Wang Z.L. (2019). Multifunctional Sensor Based on Translational-Rotary Triboelectric Nanogenerator. Adv. Energy Mater..

[44] Jiang Y., Dong K., An J., Liang F., Yi J., Peng X., Ning C., Ye C., Wang Z.L. (2021). UV-Protective, Self-Cleaning, and Antibacterial Nanofiber-Based Triboelectric Nanogenerators for Self-Powered Human Motion Monitoring. ACS Appl. Mater. Interfaces.

[45] Yin J., Li J., Ramakrishna S., Xu L. (2023). Hybrid-Structured Electrospun Nanofiber Membranes as Triboelectric Nanogenerators for Self-Powered Wearable Electronics. ACS Sustain. Chem. Eng..

[46] Jao Y.T., Yang P.K., Chiu C.M., Lin Y.J., Chen S.W., Choi D., Lin Z.H. (2018). A Textile-Based Triboelectric Nanogenerator with Humidity-Resistant Output Characteristic and Its Applications in Self-Powered Healthcare Sensors. Nano Energy.

[47] Kwak S.S., Kim H., Seung W., Kim J., Hinchet R., Kim S.W. (2017). Fully Stretchable Textile Triboelectric Nanogenerator with Knitted Fabric Structures. ACS Nano.

[48] Feng M., Wu Y., Feng Y., Dong Y., Liu Y., Peng J., Wang N., Xu S., Wang D. (2022). Highly Wearable, Machine-Washable, and Self-Cleaning Fabric-Based Triboelectric Nanogenerator for Wireless Drowning Sensors. Nano Energy.

[49] Sun N., Wang G.G., Zhao H.X., Cai Y.W., Li J.Z., Li G.Z., Zhang X.N., Wang B.L., Han J.C., Wang Y. (2021). Waterproof, Breathable and Washable Triboelectric Nanogenerator Based on Electrospun Nanofiber Films for Wearable Electronics. Nano Energy.

[50] Zhang Z., Cai J. (2021). High Output Triboelectric Nanogenerator Based on PTFE and Cotton for Energy Harvester and Human Motion Sensor. Curr. Appl. Phys..

[51] Liu J., Wang J., Wang Y., Wu Z., Sun H., Yang Y., Zhang L., Kou X., Li P., Kang W. (2024). A High-Performance Stretchable Triboelectric Nanogenerator Based on Polytetrafluoroethylene (PTFE) Particles. Energy Environ. Mater..

[52] Thakur D., Seo S., Hyun J. (2023). Three-Dimensional Triboelectric Nanogenerator with Carboxymethylated Cellulose Nanofiber and Perfluoroalkoxy Films. J. Ind. Eng. Chem..

[53] Cui S., Liu D., Yang P., Liu J., Gao Y., Zhao Z., Zhou L., Zhang J., Wang Z.L., Wang J. (2023). Triboelectric-Material-Pairs Selection for Direct-Current Triboelectric Nanogenerators. Nano Energy.

[54] Sala de Medeiros M., Chanci D., Moreno C., Goswami D., Martinez R.V. (2019). Waterproof, Breathable, and Antibacterial Self-Powered e-Textiles Based on Omniphobic Triboelectric Nanogenerators. Adv. Funct. Mater..

[55] Adeogun A.O., Chukwuka A.V., Ibor O.R., Asimakopoulos A.G., Zhang J., Arukwe A. (2024). Occurrence, Bioaccumulation and Trophic Dynamics of per- and Polyfluoroalkyl Substances in Two Tropical Freshwater Lakes. Environ. Pollut..

[56] Grandjean P., Andersen E.W., Budtz-Jørgensen E., Nielsen F., Mølbak K., Weihe P., Heilmann C. (2012). Serum Vaccine Antibody Concentrations in Children Exposed to Perfluorinated Compounds. JAMA.

[57] National Academies of Sciences, Engineering, and Medicine, Health and Medicine Division, Division on Earth and Life Studies, Board on Population Health and Public Health Practice, Board on Environmental Studies and Toxicology, Committee on the Guidance on PFAS Testing and Health Outcomes (2022). Guidance on PFAS Exposure, Testing, and Clinical Follow-Up.

[58] Rankin K., Mabury S.A., Jenkins T.M., Washington J.W. (2016). A North American and Global Survey of Perfluoroalkyl Substances in Surface Soils: Distribution Patterns and Mode of Occurrence. Chemosphere.

[59] Teunen L., Bervoets L., Belpaire C., De Jonge M., Groffen T. (2021). PFAS Accumulation in Indigenous and Translocated Aquatic Organisms from Belgium, with Translation to Human and Ecological Health Risk. Environ. Sci. Eur..

[60] Sunderland E.M., Hu X.C., Dassuncao C., Tokranov A.K., Wagner C.C., Allen J.G. (2019). A Review of the Pathways of Human Exposure to Poly- and Perfluoroalkyl Substances (PFASs) and Present Understanding of Health Effects. J. Expo. Sci. Environ. Epidemiol..

[61] Lindh C.H., Rylander L., Toft G., Axmon A., Rignell-Hydbom A., Giwercman A., Pedersen H.S., Góalczyk K., Ludwicki J.K., Zvyezday V. (2012). Blood Serum Concentrations of Perfluorinated Compounds in Men from Greenlandic Inuit and European Populations. Chemosphere.

[62] Hu X.C., Andrews D.Q., Lindstrom A.B., Bruton T.A., Schaider L.A., Grandjean P., Lohmann R., Carignan C.C., Blum A., Balan S.A. (2016). Detection of Poly- and Perfluoroalkyl Substances (PFASs) in U.S. Drinking Water Linked to Industrial Sites, Military Fire Training Areas, and Wastewater Treatment Plants. Environ. Sci. Technol. Lett..

[63] Begley T.H., Hsu W., Noonan G., Diachenko G. (2008). Migration of Fluorochemical Paper Additives from Food-Contact Paper into Foods and Food Simulants. Food Addit. Cont.—Part. A.

[64] Ragnarsdóttir O., Abou-Elwafa Abdallah M., Harrad S. (2024). Dermal Bioavailability of Perfluoroalkyl Substances Using in Vitro 3D Human Skin Equivalent Models. Environ. Int..

[65] Ragnarsdóttir O., Abdallah M.A.E., Harrad S. (2022). Dermal Uptake: An Important Pathway of Human Exposure to Perfluoroalkyl Substances?. Environ. Pollut..

[66] Lohmann R., Cousins I.T., Dewitt J.C., Glüge J., Goldenman G., Herzke D., Lindstrom A.B., Miller M.F., Ng C.A., Patton S. (2020). Are Fluoropolymers Really of Low Concern for Human and Environmental Health and Separate from Other PFAS?. Environ. Sci. Technol..

[67] Capillo G., Savoca S., Panarello G., Mancuso M., Branca C., Romano V., D’Angelo G., Bottari T., Spanò N. (2020). Quali-Quantitative Analysis of Plastics and Synthetic Microfibers Found in Demersal Species from Southern Tyrrhenian Sea (Central Mediterranean). Mar. Pollut. Bull..

[68] Zheng T., Li G., Zhang L., Lei Y. (2024). Breathable, Transparent, Waterproof, Flexible and High-Output Triboelectric Nanogenerators for Sport Monitoring and Speech Recognition. J. Mater. Chem. A Mater..

[69] Tian X., Hua T. (2021). Antibacterial, Scalable Manufacturing, Skin-Attachable, and Eco-Friendly Fabric Triboelectric Nanogenerators for Self-Powered Sensing. ACS Sustain. Chem. Eng..

[70] Zhao Z., Yan C., Liu Z., Fu X., Peng L.M., Hu Y., Zheng Z. (2016). Machine-Washable Textile Triboelectric Nanogenerators for Effective Human Respiratory Monitoring through Loom Weaving of Metallic Yarns. Adv. Mater..

[71] Ahmed A., Hassan I., Mosa I.M., Elsanadidy E., Phadke G.S., El-Kady M.F., Rusling J.F., Selvaganapathy P.R., Kaner R.B. (2019). All Printable Snow-Based Triboelectric Nanogenerator. Nano Energy.

[72] Peng F., Liu D., Zhao W., Zheng G., Ji Y., Dai K., Mi L., Zhang D., Liu C., Shen C. (2019). Facile Fabrication of Triboelectric Nanogenerator Based on Low-Cost Thermoplastic Polymeric Fabrics for Large-Area Energy Harvesting and Self-Powered Sensing. Nano Energy.

[73] Yang H., Zhang S., Li J. (2024). A Self-Powered Triboelectric Sensor for Basketball Monitoring. AIP Adv..

[74] Zhang H., Hao Q., Liu H. (2024). A Flat-Structured Triboelectric Nanogenerator Based on PDMS/MXene for Mechanical Energy Harvesting Boxing Training Monitoring. AIP Adv..

[75] Anwer S., Umair Khan M., Mohammad B., Rezeq M., Cantwell W., Gan D., Zheng L. (2023). Engineering of Electrodes with 2D Ti3C2Tx-MXene Sheets and Chloride Salt for Robust and Flexible High Electrical Power Triboelectric Nanogenerator. Chem. Eng. J..

[76] Kim I., Jeon H., Kim D., You J., Kim D. (2018). All-in-One Cellulose Based Triboelectric Nanogenerator for Electronic Paper Using Simple Filtration Process. Nano Energy.

[77] Nie S., Fu Q., Lin X., Zhang C., Lu Y., Wang S. (2021). Enhanced Performance of a Cellulose Nanofibrils-Based Triboelectric Nanogenerator by Tuning the Surface Polarizability and Hydrophobicity. Chem. Eng. J..

[78] Fang Z., Lou W., Zhang W., Guan X., He J., Lin J. (2023). Modulating Crystallinity and Dielectric Constant of Chitosan Film for Triboelectric Polarity Shift and Performance Enhancement in Triboelectric Nanogenerators. Nano Energy.

[79] Fang Z., Guan X., He J. (2025). Effectively Altering the Triboelectric Charging Behavior of Chitosan Films via Tannic Acid Surface Modification. Nano Energy.

[80] Lin Z., Yang J., Li X., Wu Y., Wei W., Liu J., Chen J., Yang J. (2018). Large-Scale and Washable Smart Textiles Based on Triboelectric Nanogenerator Arrays for Self-Powered Sleeping Monitoring. Adv. Funct. Mater..

[81] Mudgal T., Tiwari M., Bharti D. (2022). Polystyrene-Based Triboelectric Nanogenerators for Self-Powered Multifunctional Human Activity Monitoring. ACS Appl. Energy Mater..

[82] Li G.Z., Wang G.G., Cai Y.W., Sun N., Li F., Zhou H.L., Zhao H.X., Zhang X.N., Han J.C., Yang Y. (2020). A High-Performance Transparent and Flexible Triboelectric Nanogenerator Based on Hydrophobic Composite Films. Nano Energy.

[83] Park H.W., Huynh N.D., Kim W., Hwang H.J., Hong H., Choi K.H., Song A., Chung K.B., Choi D. (2018). Effects of Embedded TiO2-x Nanoparticles on Triboelectric Nanogenerator Performance. Micromachines.

[84] Chen J., Guo H., He X., Liu G., Xi Y., Shi H., Hu C. (2016). Enhancing Performance of Triboelectric Nanogenerator by Filling High Dielectric Nanoparticles into Sponge PDMS Film. ACS Appl. Mater. Interfaces.

[85] Li W., Xiang Y., Zhang W., Loos K., Pei Y. (2023). Ordered Mesoporous SiO2 Nanoparticles as Charge Storage Sites for Enhanced Triboelectric Nanogenerators. Nano Energy.

[86] Vafaiee M., Ejehi F., Mohammadpour R. (2023). CNT-PDMS Foams as Self-Powered Humidity Sensors Based on Triboelectric Nanogenerators Driven by Finger Tapping. Sci. Rep..

[87] Lee K., Mhin S., Han H.S., Kwon O., Kim W.B., Song T., Kang S., Kim K.M. (2022). A High-Performance PDMS-Based Triboelectric Nanogenerator Fabricated Using Surface-Modified Carbon Nanotubes. J. Mater. Chem. A Mater..

[88] Zhang X.Q., Yin L.H., Tang M., Pu Y.P. (2011). ZnO, TiO_2_, SiO_2_, and Al_2_O_3_ Nanoparticles-Induced Toxic Effects on Human Fetal Lung Fibroblasts. Biomed. Environ. Sci..

[89] Busolo T., Ura D.P., Kim S.K., Marzec M.M., Bernasik A., Stachewicz U., Kar-Narayan S. (2019). Surface Potential Tailoring of PMMA Fibers by Electrospinning for Enhanced Triboelectric Performance. Nano Energy.

[90] Parandeh S., Kharaziha M., Karimzadeh F. (2019). An Eco-Friendly Triboelectric Hybrid Nanogenerators Based on Graphene Oxide Incorporated Polycaprolactone Fibers and Cellulose Paper. Nano Energy.

[91] Naguib M., Mochalin V.N., Barsoum M.W., Gogotsi Y. (2014). 25th Anniversary Article: MXenes: A New Family of Two-Dimensional Materials. Adv. Mater..

[92] Mohan R., Ali F. (2023). The Future of Energy Harvesting: A Brief Review of MXenes-Based Triboelectric Nanogenerators. Polym. Adv. Technol..

[93] Habib T., Zhao X., Shah S.A., Chen Y., Sun W., An H., Lutkenhaus J.L., Radovic M., Green M.J. (2019). Oxidation Stability of Ti3C2Tx MXene Nanosheets in Solvents and Composite Films. NPJ 2D Mater. Appl..

[94] Amani A.M., Tayebi L., Vafa E., Abbasi M., Vaez A., Kamyab H., Chelliapan S., Azizli M.J., Bazargan-Lari R. (2024). On the Horizon of Greener Pathways to Travel into a Greener Future Portal: Green MXenes, Environment-Friendly Synthesis, and Their Innovative Applications. J. Clean. Prod..

[95] Saxena S., Johnson M., Dixit F., Zimmermann K., Chaudhuri S., Kaka F., Kandasubramanian B. (2023). Thinking Green with 2-D and 3-D MXenes: Environment Friendly Synthesis and Industrial Scale Applications and Global Impact. Renew. Sustain. Energy Rev..

[96] Sun W., Liu X., Hua W., Wang S., Wang S., Yu J., Wang J., Yong Q., Chu F., Lu C. (2023). Self-Strengthening and Conductive Cellulose Composite Hydrogel for High Sensitivity Strain Sensor and Flexible Triboelectric Nanogenerator. Int. J. Biol. Macromol..

[97] Zou Y., Tan P., Shi B., Ouyang H., Jiang D., Liu Z., Li H., Yu M., Wang C., Qu X. (2019). A Bionic Stretchable Nanogenerator for Underwater Sensing and Energy Harvesting. Nat. Commun..

[98] Shi Y., Wei X., Wang K., He D., Yuan Z., Xu J., Wu Z., Wang Z.L. (2021). Integrated All-Fiber Electronic Skin toward Self-Powered Sensing Sports Systems. ACS Appl. Mater. Interfaces.

[99] Tan D., Xu B., Gao Y., Tang Y., Liu Y., Yang Y., Li Z. (2022). Breathable Fabric-Based Triboelectric Nanogenerators with Open-Porous Architected Polydimethylsiloxane Coating for Wearable Applications. Nano Energy.

[100] Gogurla N., Roy B., Park J.Y., Kim S. (2019). Skin-Contact Actuated Single-Electrode Protein Triboelectric Nanogenerator and Strain Sensor for Biomechanical Energy Harvesting and Motion Sensing. Nano Energy.

[101] Patel D., Shah D., Shah M. (2020). The Intertwine of Brain and Body: A Quantitative Analysis on How Big Data Influences the System of Sports. Ann. Data Sci..

[102] Seçkin A.Ç., Ateş B., Seçkin M. (2023). Review on Wearable Technology in Sports: Concepts, Challenges and Opportunities. Appl. Sci..

[103] Zhao T., Fu Y., Sun C., Zhao X., Jiao C., Du A., Wang Q., Mao Y., Liu B. (2022). Wearable Biosensors for Real-Time Sweat Analysis and Body Motion Capture Based on Stretchable Fiber-Based Triboelectric Nanogenerators. Biosens. Bioelectron..

[104] Wen Z., Yang Y., Sun N., Li G., Liu Y., Chen C., Shi J., Xie L., Jiang H., Bao D. (2018). A Wrinkled PEDOT:PSS Film Based Stretchable and Transparent Triboelectric Nanogenerator for Wearable Energy Harvesters and Active Motion Sensors. Adv. Funct. Mater..

[105] Yang X. (2023). A Novel Triboelectric Nanogenerator Based on PDMS/Carbon for Energy Harvesting and Long-Distance Running Monitoring. J. Electron. Mater..

[106] Lin Z., Wu Z., Zhang B., Wang Y.C., Guo H., Liu G., Chen C., Chen Y., Yang J., Wang Z.L. (2019). A Triboelectric Nanogenerator-Based Smart Insole for Multifunctional Gait Monitoring. Adv. Mater. Technol..

[107] Chang S., Liu F., Chen J., Xia L., Zhou H., Jiang J., Dong K., Zhang C., Wu Y., Chen J. (2024). An Epidermal Electrode Based Triboelectric Walking Energy Harvester for Wearable Wireless Sensing Applications. Sci. China Technol. Sci..

[108] Fan W., He Q., Meng K., Tan X., Zhou Z., Zhang G., Yang J., Lin Wang Z. (2020). Machine-Knitted Washable Sensor Array Textile for Precise Epidermal Physiological Signal Monitoring. Sci. Adv..

[109] Wu W., Wen S., Wei Y., Ruan L., Li F., Cao X., Wang Z.L., Zhang L. (2023). A Volatile Organic Compound Free Unibody Triboelectric Nanogenerator and Its Application as a Smart Green Track. Nano Energy.

[110] Sahu M., Hajra S., Panda S., Rajaitha M., Panigrahi B.K., Rubahn H.G., Mishra Y.K., Kim H.J. (2022). Waste Textiles as the Versatile Triboelectric Energy-Harvesting Platform for Self-Powered Applications in Sports and Athletics. Nano Energy.

[111] Xu J., Wei X., Li R., Shi Y., Peng Y., Wu Z., Wang Z.L. (2022). Intelligent Self-Powered Sensor Based on Triboelectric Nanogenerator for Take-off Status Monitoring in the Sport of Triple-Jumping. Nano Res..

[112] Du T., Chen Z., Dong F., Cai H., Zou Y., Zhang Y., Sun P., Xu M. (2024). Advances in Green Triboelectric Nanogenerators. Adv. Funct. Mater..

[113] Lv Q., Ma X., Zhang C., Han J., He S., Liu K., Jiang S. (2024). Nanocellulose-Based Nanogenerators for Sensor Applications: A Review. Int. J. Biol. Macromol..

